# Neoadjuvant olaparib targets hypoxia to improve radioresponse in a homologous recombination-proficient breast cancer model

**DOI:** 10.18632/oncotarget.20936

**Published:** 2017-09-15

**Authors:** Gerben R. Borst, Ramya Kumareswaran, Hatice Yücel, Seyda Telli, Trevor Do, Trevor McKee, Gaetano Zafarana, Jos Jonkers, Marcel Verheij, Mark J. O’Connor, Sven Rottenberg, Robert G. Bristow

**Affiliations:** ^1^ Princess Margaret Cancer Centre, University Health Network, Toronto, Canada; ^2^ Departments of Medical Biophysics and Radiation Oncology, University of Toronto, Toronto, Canada; ^3^ Netherlands Cancer Institute – Antoni van Leeuwenhoek Hospital, Department of Radiation Oncology, Amsterdam, The Netherlands; ^4^ Netherlands Cancer Institute – Antoni van Leeuwenhoek Hospital, Department of Molecular Biology, Amsterdam, The Netherlands; ^5^ Oncology, Innovative Medicines and Early Development, AstraZeneca, Cambridge, United Kingdom; ^6^ Institute of Animal Pathology, Vetsuisse Faculty, University of Bern, Bern, Switzerland

**Keywords:** neoadjuvant treatment, targeted therapy, radiotherapy, hypoxia, olaparib

## Abstract

Clinical trials are studying the benefits of combining the PARP-1 inhibitor olaparib with chemotherapy and radiotherapy treatment in a variety of cancer increasing the therapeutic ratio for olaparib may come from its ability to modify the tumour microenvironment by targeting homologous recombination-deficient, hypoxic tumour clonogens, and/or increasing tumour-associated vasodilation to improve oxygenation. Herein, we investigated the effect of prolonged neoadjuvant exposure to olaparib on the tumor microenvironment using a genetically-engineered mouse p53−/− syngeneic breast cancer model, which is proficient in homology-directed DNA repair. We observed increased *in vivo* growth delay and decreased *ex vivo* clonogenic survival following pre-treatment with olaparib 50 mg/kg bid Olaparib for 7 days ending 48 hours prior to a radiation dose of 12Gy. This increased *in vivo* radioresponse was associated with a decreased hypoxic fraction. This study suggests that the radiation response in patients can be improved with limited toxicity if olaparib is given in a purely neoadjuvant setting to modify the tumor microenviroment prior to the start of the radiotherapy treatment. Consequently a significant gain can be achieved in therapeutic window and clinical studies are needed to confirm this preclinical data.

## INTRODUCTION

Majority of the cancer patients undergo radiation therapy as a primary or adjuvant treatment for their cancers but may fail due to tumour clonogen radioresistance leading to poor local control and secondary metastases. Tumour specific inhibition of the DNA damage response pathway and targeting intratumoural hypoxia are two attractive strategies designed to improve the radiation response [1, 2]. PARP-1 inhibitors are of specific interest because they target the DNA repair pathway and they may also have an effect on the tumor vasculature [3–7]. PARP-1 inhibitors are now FDA-approved for advanced hereditary BRCA-mutated ovarian cancers given their ability to kill homologous recombination repair (HRR)-deficient tumour cells by synthetic lethality. Olaparib (AZD2281) is a potent inhibitor of PARP-1 and is currently being tested in the clinic in combination with radiotherapy for many tumor indications (breast, lung, prostate, pancreatic and head & neck cancers) [8]. PARP-1 is involved in many cellular processes (e.g. DNA repair, genomic stability, inflammation, differentiation, cell death [9]) and it has been shown that PARP-1 inhibition targets hypoxia-mediated repair defects in tumor cells [10] and it reduces vasculogenic mimicry [11]. Also, many current generation PARP-1 inhibitors carry a nicotinamide-related structure that can lead to vasodilation which may potentially improve the radiation response [6]. Additionally, we have shown that hypoxia can lead to decreased HRR *in vitro* leading to an acquired “BRCAness” which translated into increased sensitivity to PARP-1 inhibition in hypoxic tumour cells. This “contextual” synthetic lethality as the tumour cell kill *in vitro* was associated with an effect of the microenvironment rather than an innate genetic susceptibility, per se. However, whether the effect of prolonged PARP-1 inhibitor exposure leads to a reciprocal modification of the tumour microenvironment has not been studied.

Genetically-engineered syngeneic mouse models (GEMM) are useful to study the effects of cancer treatment within a complex tumour microenvironment given the intact immune system and the presence of syngeneic host vasculature and stroma. In this study, we report the effects of neoadjuvant olaparib prior to radiotherapy in a BRCAwt/p53null breast cancer GEMM. We show that repetitive olaparib exposure alone can result in a significantly decreased hypoxic fraction and increased tumor vascular density. These changes contributed to an improved radiation response that is independent from the inhibitory effects on the repair of exogenous DNA damage.

## RESULTS

### Neoadjuvant olaparib increases growth delay in irradiated tumors

**Figure 1:**
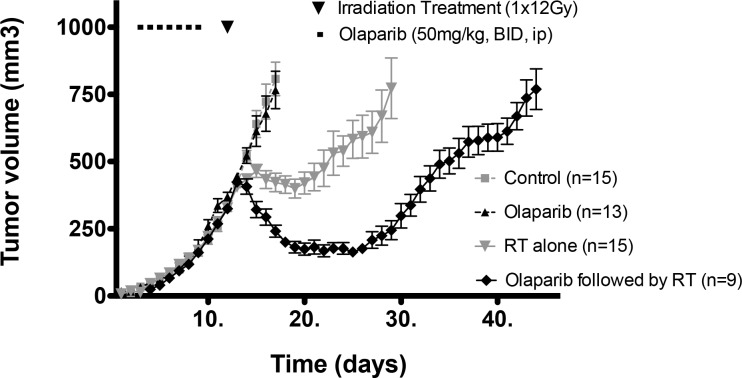
Growth curves of mice treated with vehicle (grey square), Olaparib alone (6-7 days, 50mg/kg, BID, i. p.) (black triangle), radiotherapy (RT) (grey triangle) alone (1 X 12 Gy) and Olaparib (6-7 days, 50mg/kg, BID, i.p.) followed by RT (1 X 12 Gy) (black diamond)

Previously it has been shown that interference with DNA repair caused by PARP-1 inhibitors can result in radiosensitization of tumor cells when given concurrently with radiation *in vitro* and *in vivo* [12–14]. In addition, the vasodilatative effect of olaparib and the reduction of the vascular mimicry may be a further mechanism for increased radioresponse [6, 11]. However, in this study, we wished to determine whether repetitive administration of a PARP-1 inhibitor (olaparib) prior to radiotherapy (i.e. strictly neoadjuvant, rather than concurrent, olaparib treatment) could improve tumour oxygenation prior to radiation treatment. We first investigated whether neoadjuvant olaparib would increase tumour growth delay following a drug-wash out prior to irradiation *in vivo*. We observed that tumor growth was not affected when the mice were treated with 50mg/kg BID i.p. olaparib for 6 or 7 days (Figure 1 and Supplementary Figure 1).

However, tumours treated purely neoadjuvantly with olaparib had significantly increased growth delay when followed by single dose radiotherapy (12 Gy) as compared to the growth delay following radiotherapy alone (p<0.05) (Figure 1).

**Figure 2:**
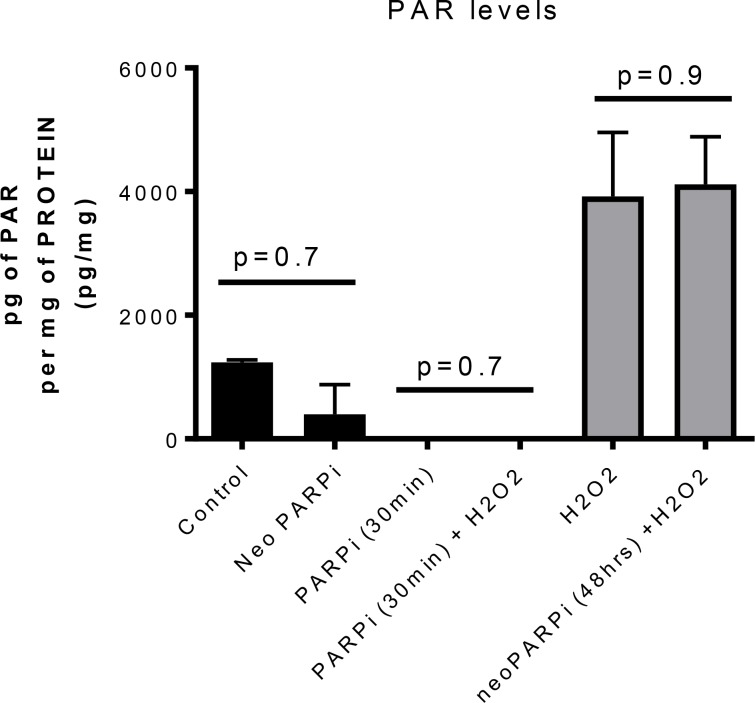
PARP activity as measured by PAR levels in three independent tumors per cohort Tumors were treated with or without H2O2 as indicated and with or without olaparib as indicated. PAR levels were increased after H2O2 injection. This increase was inhibited when olaparib was administered 30 minutes before H2O2 injection. However, if the mice were treated for 6 or 7 days with BID, 50mg/kg ip olaparib whereby the last administration was given 48 hours before the injection of H2O2, the PAR levels were not affected. No statistical difference were observed between the Control and Neoadjuvantly treated animals.

As PARP-1 inhibitors can inhibit the repair of exogenous DNA damage when given concurrently with radiotherapy (RT), we tested whether the improved growth delay with neoadjuvant olaparib treatment was dependent or independent of DNA repair inhibition. First, we used a 48h time interval between the last olaparib administration and radiotherapy as a wash-out period that previously led to undetectable olaparib drug levels in our GEMM breast cancer model [15]. Second, we measured the increase of PAR levels 48 hours after the last olaparib administration. For this, we exposed the tumors to intra-tumoral injection of H2O2, a potent single strand damaging agent (provoking PARylation) that activates PAR, as a positive control *in vivo*. When olaparib was administered to the mice 30 minutes prior to H2O2 injection, no tumour increase in PAR was observed. In contrast, following the 48h olaparib washout period, H2O2 treatment increased PAR levels significantly, and we did not observe a difference in PAR levels in olaparib-treated tumors compared to sham-injected control tumors (Figure 2). We therefore conclude that the observed increases in tumour growth delay was not caused by inhibition of PARylation by olaparib during subsequent radiation treatment.

### Tumour clonogen kill following neoadjuvant olaparib and RT is dependent on the microenvironment

**Figure 3:**
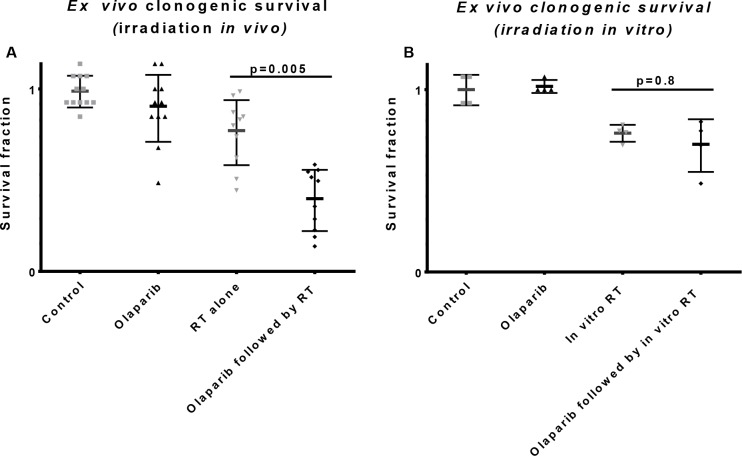
Influence of the tumor microenvironment on radiation response **(A)** Tumors were treated with vehicle (control) or olaparib (6 or 7 days, 50mg/kg, BID, ip) *in vivo* followed by mock irradiation or one fraction of 12 Gy. Twenty-four hours later tumors were harvested and plated as single cell suspension. Clonogenic survival was analyzed on the 11th day after plating with an increased clonogenic cell kill in the neoadjuvantly treated tumors. **(B)** Tumors were treated with vehicle (control) or olaparib (6 or 7 days, 50mg/kg, BID, ip) *in vivo* followed by harvesting, plating and *in vitro* mock or 12 Gy dose of radiation. There was no significant difference in radiation response between the control and neoadjuvantly treated tumors 11 days after plating.

To evaluate whether the improved irradiation response was caused by the microenvironment we compared the tumour clonogen cell kill after either in situ irradiation or ex vivo irradiation. After the in situ irradiation the tumors were harvested and plated for evaluation of clonogenic cell kill and for the ex vivo irradiation the tumors were first harvested and irradiated after plating the single cell suspension. Tumour clonogenic cell kill was only increased in neoadjuvantly olaparib treated tumors when the irradiation was performed *in vivo* (Figure 3A) and was not observed when the irradiation was given ex vivo (Figure 3B). In other words, excluding the tumour microenvironment effect before irradiation prevented the effect of neoadjuvant olaparib treatment. We conclude that the observed increase in tumor growth delay was therefore dependent on the olaparib-induced changes on the microenvironment.

### Decreased hypoxic fraction in tumors following olaparib treatment

**Figure 4:**
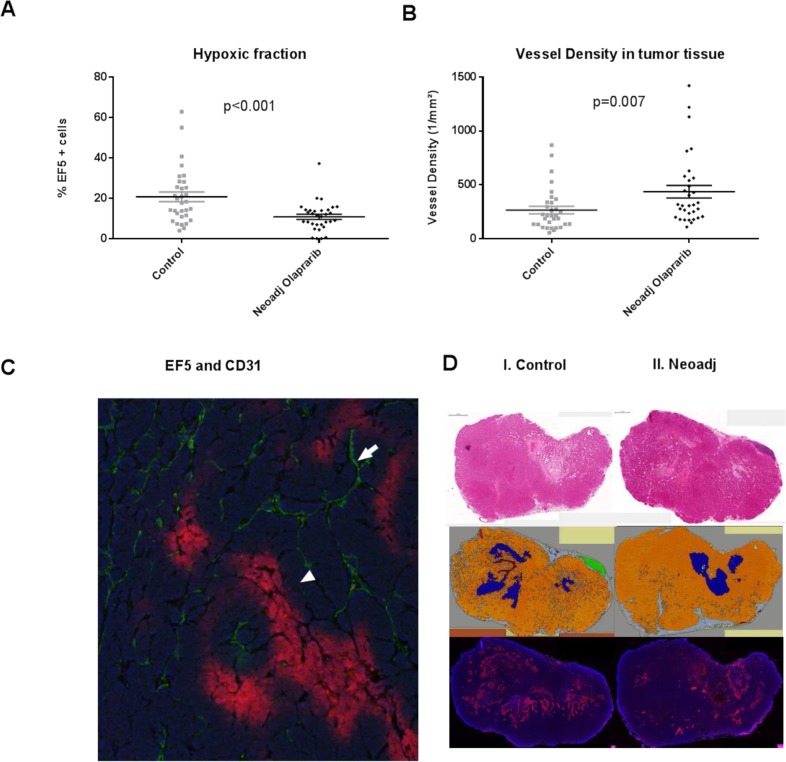
**(A)** The percentage of cells which are EF5 positive (i. e. red regions are hypoxic in Figure 4C) is significantly lower following neoadjuvant Olaparib treatment (black diamonds) when compared to the untreated control tumors (grey triangles). **(B)** The vessel density in olaparib (black diamonds) treated and control tumors (grey triangles) are different in the oxic regions within the tumor. **(C)** Hypoxic (positive EF5) area indicated by the white triangle and CD31 (vessel) is indicated by the white arrow. **(D)** I. Two H&E staining slides of a control (left) and neoadjuvantly (right) treated tumor of about 9 mm in length (scale bar of 1 mm is picture in the left top corner). H&E staining was executed to discriminate the different tissue types in the tumor. II. Discrimination: orange=tumor tissue, blue=necrotic tissue, green=normal breast tissue, brown=artefact, grey=mesenchymal tissue. III. EF5 staining of a control (left) and neoadjuvantly (right) treated tumor.

To evaluate the oxygenation status of the tumors the hypoxia tracer, EF5, was injected i.p. before harvesting the tumors (see materials and methods). To ensure a robust comparison, we also analysed the necrotic and normal tissue within the tumors (see materials and methods). The tumors were harvested 48 hours after the last olaparib injection to prevent any acute vasodilatation effect [6]. Quantification of the EF5 staining revealed that the hypoxic fraction in the neoadjuvant olaparib treated tumors was lower than the untreated control (Figure 4A). Quantification of CD31 staining revealed a higher density of vessels in the tumor tissue of the olaparib treated tumors (Figure 4B), this difference was not seen in the stromal tissue (Supplementary Figure 2). The improved oxygenation of the neoadjuvantly treated tumors resulted in more irradiation induced DNA damage as quantified by phosphorylated 53BP1 (p53BP1) at 1 hour (p=0.04) and 6 hours (p=0.02) post radiotherapy (Supplementary Figure 3). To exclude pre-existing DNA damage before radiotherapy we quantified the p53BP1 foci between control tumors and neoadjuvantly treated tumors at 48 hours following the last olaparib treatment (prior to radiotherapy). We did not observe increased p53BP1 foci before radiotherapy supporting the similar clonogenic cell kill between the control and the unirradiated neoadjuvantly treated tumors (Supplementary Figure 3). Similar to our previous finding that hypoxia leads to increased sensitivity to PARP-1 inhibition in hypoxic tumour cells (due to decreased HRR) [10], in this tumor model we observed an increased Caspase 3 expression in hypoxic regions after olaparib treatment (Supplementary Figure 4).

## DISCUSSION

In this study, we show a unique effect in that neoadjuvant PARP inhibitor olaparib had a major and permanent impact on the microenvironment leading to improved oxygenation and therefore, increased clonogen cell kill following radiotherapy. Targeted therapies are developed to treat cancer with better selectivity and efficacy in preselected patient cohorts. One of those promising strategies used genetic synthetic lethality with the inhibition of the enzyme poly (ADP-ribose) polymerase (PARP) by small molecule inhibitors in tumors which have a defect in the homologous recombination pathway, most characteristically due to BRCA mutations [16, 17]. One of the proposed mechanisms for our observation using neoadjuvant olaparib is that this manoeuvre takes advantage of a concept called contextual synthetic lethality whereby hypoxia decreased the translation and function of HR in hypoxic clonogens and thereby making them more sensitive to PARP inhibition [10]. A decreased number of tumor clonogens at the time of radiotherapy would lead to enhanced overall clonogen kill and increased growth delay. This mechanism was supported by the higher Caspase 3 activity in the hypoxic regions of the olaparib treated tumor compared to the control tumor. Because the fraction of hypoxic cells in our fast proliferative tumors is small come we didn't expect this to affect the growth rate.

Olaparib has recently been approved for ovarian cancer therapy by the FDA (in 4^th^ line) and European commission (platinum sensitive) to treat patients with ovarian cancer resulting from hereditary BRCA1 or BRCA2 mutations. Clinical trials for olaparib in combination with radiotherapy are underway (e.g. clinical trials for toxicity and the efficacy of olaparib in combination with radiation in lung, head and neck and breast cancers [8]) after pre-clinical research showed an enhanced radiation response that was correlated to the inhibition of the DNA damage repair pathway by olaparib. [12, 18, 19]. We believe that our data in a clinically relevant model now supports clinical trials where olaparib is given neoadjuvantly before radiotherapy.

PARP-1 is important for the recognition of this DNA damage, and binds to DNA strand breaks. Once DNA-bound, PARP-1 becomes catalytically activated synthesizing PAR polymers onto itself and other repair factors (i.e. PARylation). As a result, repair proteins such as XRCC1 and DNA polymerase β (pol β) are more efficiently and rapidly recruited to sites of DNA damage. In the presence of an inhibitor, PARP-1 still binds to sites of DNA damage, but PARylation is prevented enhancing radiotherapy induced cell death. Importantly, in our study we did see that the PARylation was equal in the control tumors and the neoadjuvant olaparib treated tumors after DNA damage induction. Alternatively, PARP inhibitors potentiate the cytotoxicity of DNA damaging therapies by trapping PARP1 at the DNA damage site, a DNA damage repair inhibitory process that is not reflected by our PARylation assay. PARP trapping occurring after treatment has been studied (up to 4 hours) after concurrent administration of PARP inhibitors with DNA damaging agents [20–22]. Importantly, in our treatment setup in which the DNA damaging agent (i.e. radiotherapy) is given 48 hours after the last administration of olaparib (i.e. no drugs is available in the tumor [15]) we reasonably assume that PARP trapping cannot significantly contribute to the observed increased irradiation in this rapid proliferative tumor. In other words, the differences we observed were unlikely due to inhibition of PARylation dependent DNA repair machinery.

Hypoxia is a negative prognostic and predictive factor contributing to chemoresistance, radioresistance, angiogenesis, vasculogenesis, invasiveness, metastasis, resistance to cell death, altered metabolism, and genomic instability [23]. Many of the molecular responses of hypoxia are understood but nevertheless targeting of the hypoxic subpopulation of the tumor is difficult. Olaparib is, as most other recent developed PARP inhibitors, structurally related to nicotinamide. Nicotinamide was one of the components of the ARCON treatment strategy (accelerated radiotherapy, carbogen and nicotinamide) with vasodilatative effect targeting tumor hypoxia by preventing intermittent vascular, but this strategy was never widely introduced [24]. The vasorelaxant effect of olaparib is more potent than nicotinamide but the vascular effect of prolonged olaparib exposure is not known. The washout period we used of 48 hours excluded a direct vasodilatative effect due to the nicotinamide related structure of olaparib at the time of radiotherapy. We did show however that olaparib has a permanent effect on the tumor vessel density improving the oxygenation of the tumor. The effect of PARP-1 on the angiogenesis has been studied but with conflicting results. The most recent study of Caldini et al [25] observed that a low concentration of PARP inhibitor (3ABA) stimulated angiogenesis by decreasing the fibrinolytic activity. On the other hand some groups published contrary data. For example the group of Graziani investigated the angiogenesis in a PARP-1 knock-out mice using an *in vivo* matrigel plug assay and observed that the PARP inhibitor GPI 15427 hampered formation of tubule-like networks and impaired angiogenesis [26]. Importantly, clinical trials studying the anti-tumor effect of anti-angiogenesis agents as single agent have been negative in contrast to the clinical studies using combinations of anti-angiogenesis with chemotherapy of radiotherapy [27]. This has been explained by the observation that anti-angiogenesis treatment resulted in a “normalization” of the tumor vasculature, or a decreasing vasculogenic mimicry (VM) (which is a process independent of angiogenesis). VM was described in 1999 by Maniotis et al [28] in highly aggressive and metastatic melanoma cells as a form of highly patterned vascular channels externally lined by tumor cells, without the existence of endothelial cells. Rodriquez et al [11] showed that PARP inhibition is also involved in the process of epithelial-mesenchymal transition (EMT) [29] which is a known factor involved in radioresistance [30] and reduced VM. We indeed observed that the average vessel size indeed increased in the tumors but not in the stromal tissue after neoadjuvant treatment with olaparib although we could not confirm EMT in our model (Supplementary Figure 5). This result shows that the repetitive exposure of PARP-1 inhibition indeed improved the vascular structure specifically in tumors

In conclusion we observed a significant increase in radiotherapy response if olaparib was given in a neoadjuvant treatment schedule in a HRR proficient, immune-competent and clinically relevant tumor model. This effect was correlated with improved oxygenation of the tumor and independent from inhibition of the PARylation during radiotherapy.

## MATERIALS AND METHODS

### Tumor model

Mammary tumors were generated in K14cre;p53F/F mice, genotyped, and orthotopically transplanted into syngeneic wild-type mice as previously described [15, 31]. The onset of tumor growth was checked at least 3 times per week starting a week after tumor grafting. Mammary tumor size was determined by caliper measurements (length and width in millimeters) and tumor volume (in mm3) was calculated by using the formula: 1/2 X (length X width^2^). Animals were sacrificed when the tumor volume reached 1,500 mm3. All experimental procedures on animals were approved by the Animal Ethics Committee at the Princess Margaret Cancer Centre.

### *In vivo* treatment schedule

Olaparib (AZD2281) was prepared by diluting 50 mg/ml stocks in DMSO with 10% 2-hydroxyl-propyl-beta-cyclodextrine/PBS such that the final volume administered by i.p. injection was 10 L/g of body weight. The treatment was started when the tumor size reached 3-4 mm in dimension and was continued for 6 to 7 days bi-daily (BID). The tumors were irradiated with a single fraction of 12Gy by two orthogonal fields on a small animal dedicated high precision image-guided irradiator 48 hours after the last drug treatment [14].

### Clonogenic survival assay

Tumors were harvested and cut into small pieces and digested into single cell suspensions in collagenase IV (Gibco) and hyaluronidase (Sigma) in DMEM/F12 media. The cells were counted with trypan blue using a hemocytometer. Soft agar colony formation assay was used to analyze anchorage-independent cell growth. Plates were pre-coated with 0.6% agar diluted in DMEM/F12 media. Cells were diluted in 0.2% agar and plated in triplicates in 24-well plates. The plates were kept in a 5% CO_2_, 3% O_2_ and 37°C humidified incubator. MTT (3-(4, 5-dimethylthiazolyl-2)-2,5-diphenyltetrazolium bromide) was added on the 11^th^ day following plating and the cells were incubated overnight for staining. The cells were then imaged with a tissue dissection microscope and colonies greater than 100 μm in diameter were counted using the ImagePro software.

### PARP activity assay

PARP activity was indirectly measured by poly-ADP-ribose (PAR) levels using the HT PARP *in vivo* Pharmacodynamic Assay II kit (Trevigen) as previously described [32]. After intratumoral injection of hydrogen peroxide (H2O2), tumors were harvested and immediately snap frozen. Frozen tumors were lysed, sonicated and treated with 1% SDS. The protein concentration of tumor lysates was measured using a BCA protein assay. Tumor lysates and PAR standards were incubated overnight in wells pre-coated with anti-PAR monoclonal capture antibody. The wells were later incubated with a polyclonal rabbit PAR detecting antibody for 2 hours and then with a goat anti-rabbit IgG-HRP secondary antibody for 1 hour. A chemiluminescent HRP substrate was added immediately before readings as per manufacturer's instructions. PAR values were calculated using relative light units of the PAR standards that directly correlate with the amount of cellular PAR levels.

### Pathology staining

Tumors were harvested and fixed in 10% neutral buffered formalin for 24 hours at room temperature and then replaced with 75% alcohol. Tumors were processed in a conventional tissue processor and embedded into paraffin wax using graded alcohol, xylene and paraffin wax. Serial sections of 5 μm were cut from the paraffin embedded tumor blocks for immunohistochemical analyses.

For hypoxia and proliferation analysis, mice were injected i.p. with 2-nitroimidazol hypoxia marker EF5 (30 mg/kg) and active DNA synthesis marker EdU (10 mM), respectively, 2.5 hours prior to sacrifice. For EdU staining the Click-iT cocktail was applied for 30 min at room temperature. Primary antibodies used were as follows: PECAM-1 (Santa Cruz sc-1506), Cleaved Caspase-3 (Cell Signalling), 53BP1^Ser1778^ (Cell Signalling), Vimentin (Fitzgerald 20R-VP004), E-cadherin (BD Biosciences).

Secondary antibodies were: Alexa Fluor 488 Donkey Anti-Rabbit (Life Technologies), Biotin-Goat Anti-Guinea Pig IgG (Jackson ImmunoResearch), Biotin-Goat Anti-Mouse IgG (Invitrogen)

Images were acquired using an Olympus IX81 Spinning Disk Confocal Microscope using an PlanApo 60x/1.42 oil objective lens. A minimum of 50 cells were imaged for 53BP1^Ser1778^ analysis. Images were processed and analyzed using ImagePro Plus.

### Pathology analysis

Slides were scanned using a Tissuescope (TS4000) laser scanning system for immunofluorescent and H&E brightfield images. Images were split into individual 8 bit grey scale images using Image ProPlus. Grey scale images were loaded onto Definiens Tissue Studio for analysis. Regions of interest including tumor, necrosis, stroma, artifacts, EF5, empty space and margins were detected by creating a training ruleset. Cellular analysis was performed by nuclei detection whereby the mean intensity of nuclei in each grey channel was measured and the average mean intensity was calculated among the pool of samples. Subsequently, the threshold was calculated by the average mean intensity plus two standard deviations.

### Statistics analysis

GraphPad Prism 5.0 was used for statistical comparison between 2 groups usinga Mann-Whitney rank test. All data were expressed as mean with the standard deviation (SD) or standard error of the mean (SEM) as indicated.

## SUPPLEMENTARY FIGURES



## Abbreviations

ARCON: accelerated radiotherapy, carbogen and nicotinamide
BID: (bis in die), twice a day
BRCA: Breast Cancer
DMSO: dimethyl sulfoxide
EdU: 5-ethynyl-2′-deoxyuridine
EF5: 2-nitroimidazole
EMT: epithelial-mesenchymal transition
FDA: Food and Drug Administration
GEMM: Genetically Engineered Mouse Models
Gy: Gray
HRR: Homologous recombination Repair
MTT: 3-(4, 5-dimethylthiazolyl-2)-2,5-diphenylte-trazolium bromide
PARP: poly-ADP-ribose polymerase
VM: vasculogenic mimicry

## References

[1] Begg AC, Stewart FA, Vens C (2011). Strategies to improve radiotherapy with targeted drugs. Nat Rev Cancer.

[2] Borst GR, McLaughlin M, Kyula JN, Neijenhuis S, Khan A, Good J, Zaidi S, Powell NG, Meier P, Collins I, Garrett MD, Verheij M, Harrington KJ (2013). Targeted radiosensitization by the Chk1 inhibitor SAR-020106. Int J Radiat Oncol Biol Phys.

[3] Ben-Hur E, Utsumi H, Elkind MM (1984). Inhibitors of poly (ADP-ribose) synthesis enhance radiation response by differentially affecting repair of potentially lethal versus sublethal damage. Br J Cancer Suppl.

[4] de Murcia JM, Niedergang C, Trucco C, Ricoul M, Dutrillaux B, Mark M, Oliver FJ, Masson M, Dierich A, LeMeur M, Walztinger C, Chambon P, de Murcia G (1997). Requirement of poly (ADP-ribose) polymerase in recovery from DNA damage in mice and in cells. Proc Natl Acad Sci U S A.

[5] Molinete M, Vermeulen W, Burkle A, Menissier-de Murcia J, Kupper JH, Hoeijmakers JH, de Murcia G (1993). Overproduction of the poly (ADP-ribose) polymerase DNA-binding domain blocks alkylation-induced DNA repair synthesis in mammalian cells. EMBO J.

[6] Senra JM, Telfer BA, Cherry KE, McCrudden CM, Hirst DG, O’Connor MJ, Wedge SR, Stratford IJ (2011). Inhibition of PARP-1 by olaparib (AZD2281) increases the radiosensitivity of a lung tumor xenograft. Mol Cancer Ther.

[7] Utsumi H, Elkind MM (1983). Caffeine-enhanced survival of radiation-sensitive, repair-deficient Chinese hamster cells. Radiat Res.

[8] https://clinicaltrials.gov/ct2/show/NCT01562210.

[9] Laudisi F, Sambucci M, Pioli C (2011). Poly (ADP-ribose) polymerase-1 (PARP-1) as immune regulator. Endocr Metab Immune Disord Drug Targets.

[10] Chan N, Pires IM, Bencokova Z, Coackley C, Luoto KR, Bhogal N, Lakshman M, Gottipati P, Oliver FJ, Helleday T, Hammond EM, Bristow RG (2010). Contextual synthetic lethality of cancer cell kill based on the tumor microenvironment. Cancer Res.

[11] Rodriguez MI, Peralta-Leal A, O’Valle F, Rodriguez-Vargas JM, Gonzalez-Flores A, Majuelos-Melguizo J, Lopez L, Serrano S, de Herreros AG, Rodriguez-Manzaneque JC, Fernandez R, Del Moral RG, de Almodovar JM (2013). PARP-1 regulates metastatic melanoma through modulation of vimentin-induced malignant transformation. PLoS Genet.

[12] Dungey FA, Loser DA, Chalmers AJ (2008). Replication-dependent radiosensitization of human glioma cells by inhibition of poly (ADP-Ribose) polymerase: mechanisms and therapeutic potential. Int J Radiat Oncol Biol Phys.

[13] Liu SK, Coackley C, Krause M, Jalali F, Chan N, Bristow RG (2008). A novel poly (ADP-ribose) polymerase inhibitor, ABT-888, radiosensitizes malignant human cell lines under hypoxia. Radiother Oncol.

[14] Veuger SJ, Curtin NJ, Richardson CJ, Smith GC, Durkacz BW (2003). Radiosensitization and DNA repair inhibition by the combined use of novel inhibitors of DNA-dependent protein kinase and poly (ADP-ribose) polymerase-1. Cancer Res.

[15] Rottenberg S, Jaspers JE, Kersbergen A, van der Burg E, Nygren AO, Zander SA, Derksen PW, de Bruin M, Zevenhoven J, Lau A, Boulter R, Cranston A, O’Connor MJ (2008). High sensitivity of BRCA1-deficient mammary tumors to the PARP inhibitor AZD2281 alone and in combination with platinum drugs. Proc Natl Acad Sci U S A.

[16] Bryant HE, Schultz N, Thomas HD, Parker KM, Flower D, Lopez E, Kyle S, Meuth M, Curtin NJ, Helleday T (2005). Specific killing of BRCA2-deficient tumours with inhibitors of poly (ADP-ribose) polymerase. Nature.

[17] Farmer H, McCabe N, Lord CJ, Tutt AN, Johnson DA, Richardson TB, Santarosa M, Dillon KJ, Hickson I, Knights C, Martin NM, Jackson SP, Smith GC (2005). Targeting the DNA repair defect in BRCA mutant cells as a therapeutic strategy. Nature.

[18] Godon C, Cordelieres FP, Biard D, Giocanti N, Megnin-Chanet F, Hall J, Favaudon V (2008). PARP inhibition versus PARP-1 silencing: different outcomes in terms of single-strand break repair and radiation susceptibility. Nucleic Acids Res.

[19] Noel G, Godon C, Fernet M, Giocanti N, Megnin-Chanet F, Favaudon V (2006). Radiosensitization by the poly (ADP-ribose) polymerase inhibitor 4-amino-1,8-naphthalimide is specific of the S phase of the cell cycle and involves arrest of DNA synthesis. Mol Cancer Ther.

[20] Hopkins TA, Shi Y, Rodriguez LE, Solomon LR, Donawho CK, DiGiammarino EL, Panchal SC, Wilsbacher JL, Gao W, Olson AM, Stolarik DF, Osterling DJ, Johnson EF (2015). Mechanistic dissection of PARP1 trapping and the impact on in vivo tolerability and efficacy of PARP inhibitors. Mol Cancer Res.

[21] Murai J, Huang SY, Das BB, Renaud A, Zhang Y, Doroshow JH, Ji J, Takeda S, Pommier Y (2012). Trapping of PARP1 and PARP2 by clinical PARP inhibitors. Cancer Res.

[22] Murai J, Huang SY, Renaud A, Zhang Y, Ji J, Takeda S, Morris J, Teicher B, Doroshow JH, Pommier Y (2014). Stereospecific PARP trapping by BMN 673 and comparison with olaparib and rucaparib. Mol Cancer Ther.

[23] Wilson WR, Hay MP (2011). Targeting hypoxia in cancer therapy. Nat Rev Cancer.

[24] Kaanders JH, Bussink J, van der Kogel AJ (2002). ARCON: a novel biology-based approach in radiotherapy. Lancet Oncol.

[25] Caldini R, Fanti E, Magnelli L, Barletta E, Tanganelli E, Zampieri M, Chevanne M (2011). Low doses of 3-aminobenzamide, a poly (ADP-ribose) polymerase inhibitor, stimulate angiogenesis by regulating expression of urokinase type plasminogen activator and matrix metalloprotease 2. Vasc Cell.

[26] Tentori L, Lacal PM, Muzi A, Dorio AS, Leonetti C, Scarsella M, Ruffini F, Xu W, Min W, Stoppacciaro A, Colarossi C, Wang ZQ, Zhang J (2007). Poly (ADP-ribose) polymerase (PARP) inhibition or PARP-1 gene deletion reduces angiogenesis. Eur J Cancer.

[27] Al-Husein B, Abdalla M, Trepte M, Deremer DL, Somanath PR (2012). Antiangiogenic therapy for cancer: an update. Pharmacotherapy.

[28] Maniotis AJ, Folberg R, Hess A, Seftor EA, Gardner LM, Pe’er J, Trent JM, Meltzer PS, Hendrix MJ (1999). Vascular channel formation by human melanoma cells in vivo and in vitro: vasculogenic mimicry. Am J Pathol.

[29] Pu H, Horbinski C, Hensley PJ, Matuszak EA, Atkinson T, Kyprianou N (2014). PARP-1 regulates epithelial-mesenchymal transition (EMT) in prostate tumorigenesis. Carcinogenesis.

[30] Kurrey NK, Jalgaonkar SP, Joglekar AV, Ghanate AD, Chaskar PD, Doiphode RY, Bapat SA (2009). Snail and slug mediate radioresistance and chemoresistance by antagonizing p53-mediated apoptosis and acquiring a stem-like phenotype in ovarian cancer cells. Stem Cells.

[31] Jonkers J, Meuwissen R, van der Gulden H, Peterse H, van der Valk M, Berns A (2001). Synergistic tumor suppressor activity of BRCA2 and p53 in a conditional mouse model for breast cancer. Nat Genet.

[32] Kumareswaran R, Chaudary N, Jaluba K, Meng A, Sykes J, Borhan A, Hill RP, Bristow RG (2015). Cyclic hypoxia does not alter RAD51 expression or PARP inhibitor cell kill in tumor cells. Radiother Oncol.

