# Correction: Neoadjuvant olaparib targets hypoxia to improve radioresponse in a homologous recombination-proficient breast cancer model

**DOI:** 10.18632/oncotarget.27476

**Published:** 2020-04-28

**Authors:** Gerben R. Borst, Ramya Kumareswaran, Hatice Yücel, Seyda Telli, Trevor Do, Trevor McKee, Gaetano Zafarana, Jos Jonkers, Marcel Verheij, Mark J. O’Connor, Sven Rottenberg, Robert G. Bristow

**Affiliations:** ^1^ Princess Margaret Cancer Centre, University Health Network, Toronto, Canada; ^2^ Departments of Medical Biophysics and Radiation Oncology, University of Toronto, Toronto, Canada; ^3^ Netherlands Cancer Institute – Antoni van Leeuwenhoek Hospital, Department of Radiation Oncology, Amsterdam, The Netherlands; ^4^ Netherlands Cancer Institute – Antoni van Leeuwenhoek Hospital, Department of Molecular Biology, Amsterdam, The Netherlands; ^5^ Oncology, Innovative Medicines and Early Development, AstraZeneca, Cambridge, United Kingdom; ^6^ Institute of Animal Pathology, Vetsuisse Faculty, University of Bern, Bern, Switzerland

**This article has been corrected:** Due to errors in image processing, in the top panel of Figure 4D, the top row showing an example of 2 H&E sections of 2 tumors was accidentally switched in position. The corrected Figure 4D is shown below. The authors declare that these corrections do not change the results or conclusions of this paper.

Original article: Oncotarget. 2017; 8:87638–87646. 87638-87646. https://doi.org/10.18632/oncotarget.20936

**Figure 4:**
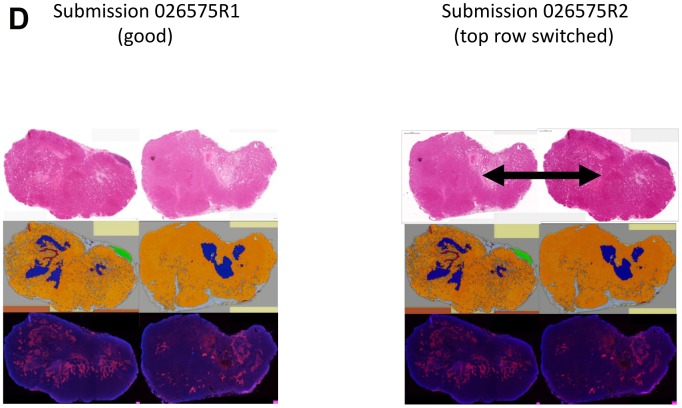
(**A**) The percentage of cells which are EF5 positive (i. e. red regions are hypoxic in Figure 4C) is significantly lower following neoadjuvant Olaparib treatment (black diamonds) when compared to the untreated control tumors (grey triangles). (**B**) The vessel density in olaparib (black diamonds) treated and control tumors (grey triangles) are different in the oxic regions within the tumor. (**C**) Hypoxic (positive EF5) area indicated by the white triangle and CD31 (vessel) is indicated by the white arrow. (**D**) I. Two H&E staining slides of a control (left) and neoadjuvantly (right) treated tumor of about 9 mm in length (scale bar of 1 mm is picture in the left top corner). H&E staining was executed to discriminate the different tissue types in the tumor. II. Discrimination: orange=tumor tissue, blue=necrotic tissue, green=normal breast tissue, brown=artefact, grey=mesenchymal tissue. III. EF5 staining of a control (left) and neoadjuvantly (right) treated tumor.

