# Supplementary material for: Neoadjuvant olaparib targets hypoxia to improve radioresponse in a homologous recombination-proficient breast cancer model
Source: Oncotarget. 2017 Sep 15;8(50):87638–46. doi: 10.18632/oncotarget.20936 (PMC5675659; doi:10.18632/oncotarget.20936)
Supplement: Supplementary file 1 [file oncotarget-08-87638-s001.pdf]

Neoadjuvant olaparib targets hypoxia to improve radioresponse in a homologous recombination-proficient breast cancer model

SUPPLEMENTARY MATERIALS

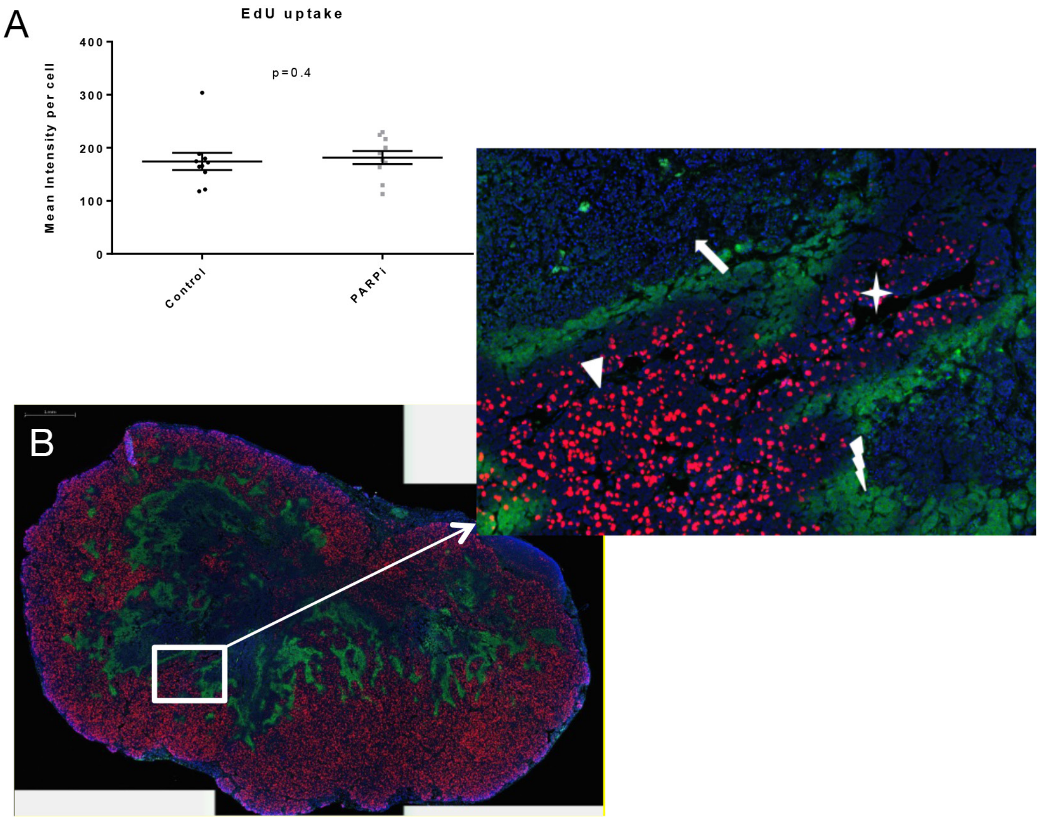

Supplementary Figure 1: (A) The uptake of EdU (purple fluorescence) was similar between olaparib treated and control tumors (p=0.4). **(B)** One example of EdU staining of control tumor (same tumor as used in figure 3); Highly proliferative area indicated by triangle, necrotic area indicated by arrow, low proliferative area indicated by star and hypoxic area indicated by lightening. The scale bar of 1 mm is indicated in the left top corner.

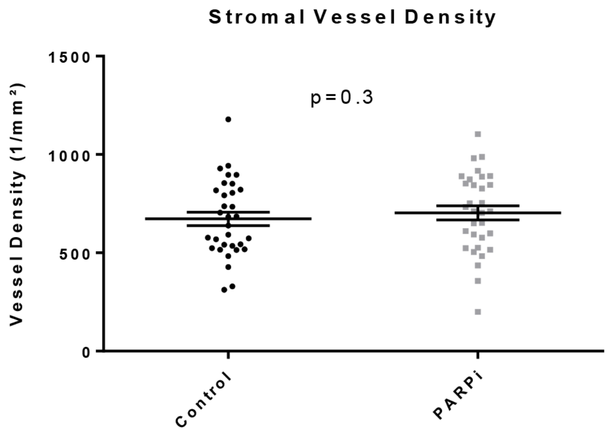

Supplementary Figure 2: There is no change in vessel density in stroma of the control and olaparib treated tumors at 48 hours after the last olaparib treatment (p=0. 3). In Figure 3 the staining of the vessel density is given.

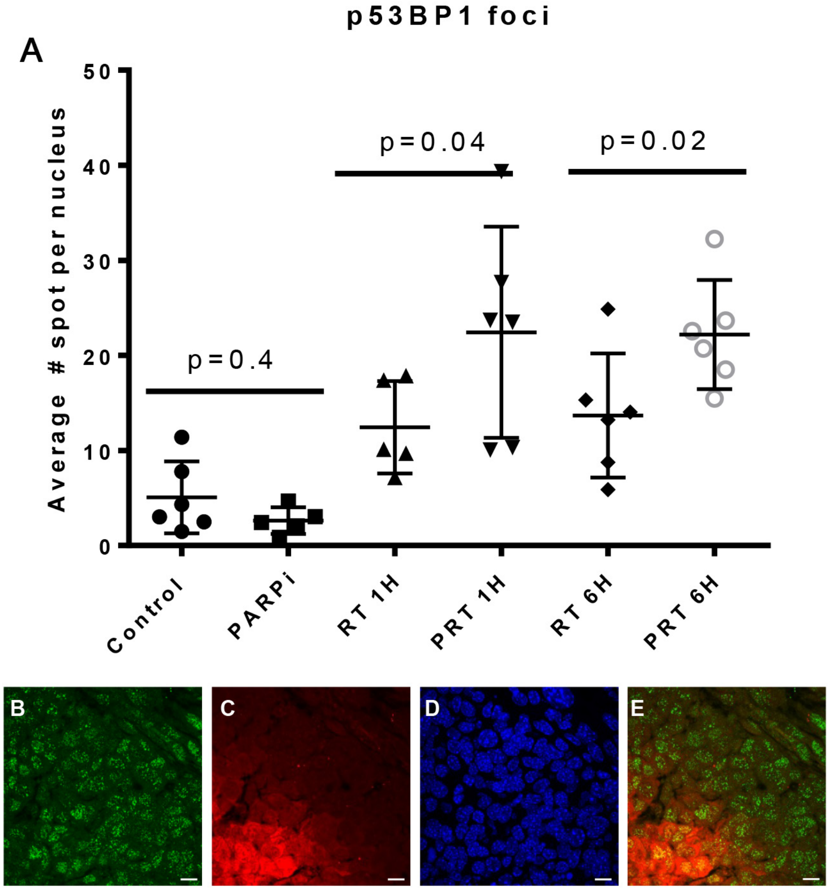

Supplementary Figure 3: (A) Quantification of p53BP1 foci in control, olaparib (PARPi), RT, and neoadjuvant olaparib plus RT (PRT) tumors with significant differences between radiotherapy alone versus radiotherapy after neoadjuvant olaparib 1 and 6 hours after irradiation. For this analysis whole tumor slides were analyzed. Immunohistochemistry examples are given for 53BP1 **(B)**, EF5 **(C)**, DAPI **(D)** and the **(E)** overlay (Scale bar = 100 um).

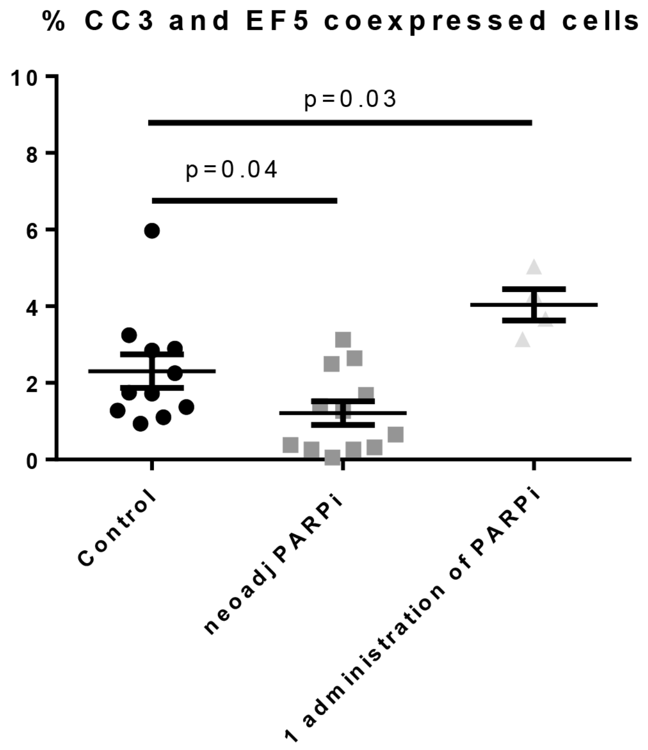

Supplementary Figure 4: Coexpression of Caspase 3 and EF5 in control (n=11), neoadjuvantly olaparib treated (n=12) and once olaparib treated mice (n=5). A significant decrease of Caspase 3 and EF 5 coexpression is observed after neoadjuvant olaparib (p=0.04) whereas the increase of coexpression after only 1 administration was increased (p=0.03).

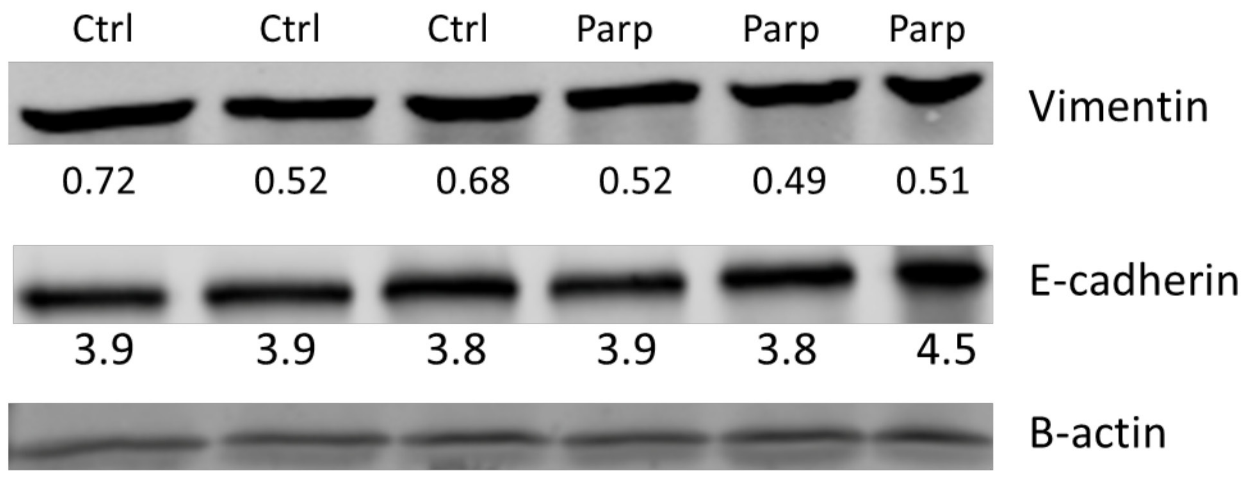

Supplementary Figure 5: There is no statistical difference in the expression of vimentin (p=0. 2) and E-cadherin (p=0.99) protein levels in in control versus neoadjuvantly olaparib treated (BID for 7 days) tumors.
